# Adverse Impact of DNA Methylation Regulatory Gene Mutations on the Prognosis of AML Patients in the 2017 ELN Favorable Risk Group, Particularly Those Defined by *NPM1* Mutation

**DOI:** 10.3390/diagnostics11060986

**Published:** 2021-05-29

**Authors:** James Yu, Jingxin Sun, Yuan Du, Rushang Patel, Juan Carlos Varela, Shahram Mori, Chung-Che Chang

**Affiliations:** 1 Department of Internal Medicine, AdventHealth Orlando, Orlando, FL 32804, USA; James.Yu.MD@AdventHealth.com; 2 Department of Hematology and Medical Oncology, Baylor College of Medicine, Houston, TX 77030, USA; Jingxin.Sun@bcm.edu; 3 Research Institute, AdventHealth Orlando Hospital, Orlando, FL 32804, USA; Yuan.Du@AdventHealth.com; 4 Blood and Marrow Transplant Center, AdventHealth Orlando Hospital, Orlando, FL 32804, USA; Rushang.Patel.MD@AdventHealth.com (R.P.); Juan.Varela.MD@AdventHealth.com (J.C.V.); Shahram.Mori.MD@AdventHealth.com (S.M.); 5 Department of Pathology and Laboratory Medicine, AdventHealth Orlando Hospital, Orlando, FL 32804, USA; 6 Department of Pathology, College of Medicine, University of Central Florida, Orlando, FL 32804, USA

**Keywords:** acute myeloid leukemia, DNA methylation regulatory gene mutations, *DNMT3A*, *IDH1/2*, NGS, *TET2*, 2017 ELN risk stratification

## Abstract

The 2017 ELN risk stratification has been widely adopted, but some studies have suggested the outcomes are heterogenous within the ELN risk groups and may be affected by other co-existing genetic mutations. This study evaluated the impact of DNA methylation regulatory gene (*TET2*, *IDH1/2*, *DNMT3A*, *SETBP1*) mutations (DMRGM) evaluated by NGS in the outcome of AML patients in each ELN risk group. A total of 114 patients were analyzed with a median follow-up of 12 months. Overall, 30.7% (35/114) of patients had DMRGM. DMRGM status had no impact on CR rate in each ELN risk group. The OS, however, was significantly shorter in patients with DMRGM compared to those without DMRGM (median OS: 12 vs. 33 months, *p =* 0.0053). Multivariate analysis showed DMRGM status was an independent unfavorable factor for OS (HR: 2.704, 95% CI: 1.451–5.041, *p =* 0.0017). The adverse OS impact of DMRGM was only observed in the ELN favorable group (7 months vs. not reached, *p =* 0.0001), but not in the intermediate or adverse group. Among the favorable group with DMRGM (n = 16), DMRGM occurred predominantly in cases with mutated *NPM1* (15/16, or 93.8%). Our results suggest that DMRGM adversely impact the outcomes of ELN favorable group patients, particularly those with mutated *NPM1*. Further studies are warranted to confirm our observations.

## 1. Introduction

Acute myeloid leukemia (AML) is a heterogeneous aggressive blood cell cancer which is the most common acute leukemia in adults [1]. Next-generation sequencing (NGS) has emerged as an important tool in the identification of mutated genes in AML [2,3,4]. Recently, large studies have identified multiple such gene mutations that significantly impact the prognostic outcomes of AML [5,6,7,8]. In 2017, the European Leukemia Net (ELN) stratified AML patients into three risk groups based on the presence or absence of specific chromosomal abnormalities and selected gene mutations [9]. As a result, the impact of *NPM1*, *FLT3*-ITD and *CEBPA* mutations was further defined based on the presence of other mutations such as *RUNX1*, *ASXL1* and *TP53*. This has advanced prognosis and therapeutic options in AML. Some studies, however, have suggested that complete remission (CR) rates and outcomes are heterogenous within the 2017 ELN risk groups and may be affected by other co-existing genetic mutations [10,11,12].

One important class of mutations in AML regulates DNA methylation patterns. Genes such as *DNMT3A*, *TET2*, *IDH1* and *IDH2*, collectively referred to here as DNA methylation regulatory gene mutations (DMRGM), have been shown to be associated with poor prognosis [5,11,13,14,15,16,17,18]. Others, however, have not shown similar prognostic impacts of such mutations of DMRGM in AML patients [19,20]. It is well established that *IDH1* and *2* are mutually exclusively in AML [21]. In addition, few studies with a large number of AML patient have demonstrated that *TET2* mutation is mutually exclusive with *IDH1* and *IDH2* mutations [18,22]. Based on these observations, it has been suggested that the mutations of DNA methylation regulatory genes may have similar biological and prognostic effects in AML [6,15,22,23]. Of note, a prior study by Ryotokuji T et al. in 2016, combining these DMRGM as one factor, revealed the unfavorable prognostic impact of DMRGM for overall survival (OS) among AML patients [24]. These observations prompted us to explore the prognostic impact of DMRGM in each group of the 2017 ELN risk group stratification.

## 2. Materials and Methods

### 2.1. Patients

Adult AML patients aged 18–75 who underwent intensive induction chemotherapy at our institution from 2017 to 2020 were screened for this study. All patients had bone marrow biopsies for morphologic evaluation, flow cytometry immunophenotyping, conventional karyotyping and next generation sequencing (NGS) study for AML-associated gene mutations at diagnosis. The diagnoses and classification of AML were based on the 2016 World Health Organization (WHO) classification of AML, and risk groups were assigned using the 2017 ELN risk stratification scheme [9,25]. Patients with treatment related-AML (t-AML) and acute promyelocytic leukemia (APL) were excluded. All patients in the study received intensive chemotherapy with or without *FLT3* inhibitors as appropriate and/or hematopoietic stem cell transplantation (HSCT) based on standard protocols. The study was approved by the institutional review board.

### 2.2. NGS Study and FLT3-ITD Measurement

The NGS study was performed using a CLIA certified laboratory developed target panel covering 53 AML related genes, including ATM, AXSL1, BCOR, BCORL1, BRAF, CBL, CEBPA, CREBBP, CSF1R, CSF3R, CSF4R, DNMT3A, EZH2, FBWX7, FGFR4, FLT3-ITD, FLT3-TKD, GATA1, GATA2, IDH1, IDH2, JAK2, KDM6A, KIT, KRAS, MPL, NF1, NOTCH2, NPM1, NRAS, PDGFRA, PDGGRB, PTPN11, RUNX1, SETBP1, SF3B1, SRSF2, STAG2, TET2, TP53, U2AF1, WT1, and ZRSR2. The panel sequenced all coding regions of the genes tested, not just known hot spots. Briefly, DNA was extracted from bone marrow aspirate or peripheral blood of each case using an Autopure extractor (Qiagen, Valencia, CA, USA) and was quantified using a Qubit DNA BR assay kit (Life Technologies, Carlsbad, CA, USA). The library was prepared using 250 ng of DNA. Target enrichment was performed using oligonucleotide-based targeted capture (xGen Lockdown Custom Target Capture Probes, Integrated DNA Technologies, and SeqCap EZ Hybridization and Wash Kit, Roche NimbleGen, Inc. Pleasanton, CA, USA) of whole genome shotgun sequencing libraries (KAPA Hyper Prep Kit and Kapa Library Amplification Kit, KAPA Biosystems, Inc. Woburn, MA, USA). Sequencing of enriched libraries was performed in multiplex on the Illumina HiSeq 2500 using the paired-end, 101 base-pair configurations. The bioinformatic analysis and annotation was performed by Clinical Genomics Workspace (PierianDX, Creve Coeur, MO, USA).

Mutations were called if the variant allele frequency (fraction) was greater than 2.5% and greater than 30 supporting reads. *FLT3*-ITD was also tested by capillary electrophoresis provided by Neogenomics Laboratory (Fort Myers, FL, USA) since NGS could miss ITD with larger base pairs and could not quantitate ITD allele fraction to determine ELN risk groups. The ITD allele fraction was determined by dividing the area under the ITD peak by the area under the wild-type allele peak.

### 2.3. Patient Group

DMRGM were defined as mutations detected in at least one of the following genes: *DNMT3A*, *TET2*, *IDH1*, *IDH2* or *SETBP1*. The patients were divided into 6 groups: groups 1 and 2 as ELN favorable group with or without DMRGM, groups 3 and 4 as ELN intermediate group with or without DMRGM, and groups 5 and 6 as ELN adverse group with or without DMRGM.

### 2.4. Statistical Analyses

The median follow-up for survival was calculated according to the Kaplan–Meier method [26]. Patient baseline characteristics between DNA methylation regulatory genes mutated or unmutated groups were performed by using the Mann–Whitney test for continuous variables and by using a chi-square test for categorical variables. The Kaplan–Meier method was used to estimate the distribution of OS in each group by the 2017 ELN risk stratification [27]. The log-rank test was used for univariate analysis to compare the OS difference between groups. Post hoc pairwise comparisons for the log-rank test with Sidak adjustment were performed to prevent type I error. The Cox proportional hazard regression model was used for multivariable analysis [28]. CR rates were compared by chi-square test or Fischer’s exact test based on sample sizes. Two-tailed statistical significance at a level of 5% was used for statistical analysis. All analyses were performed using SAS 9.4 (SAS Institute, Cary, NC, USA).

## 3. Results

### 3.1. Patients’ Characteristics

A total of 114 patients were studied (Table 1). There were 69 males (60.5%) and 45 females (39.5%) with a median age of 61.5 years (range, 19–75). The median follow-up was 12 months. Patients were nearly equally distributed among favorable, intermediate, and adverse per 2017 ELN. DMRGM were present in 30.7% (35/114) of patients. Table 1 describes the clinical and laboratory features as well as AML classification of patients by DMRGM status. There were no significant differences in age, gender, white blood cell (WBC) counts, bone marrow (BM) blasts, peripheral blood (PB) blasts, or HSCT rate between DMRGM positive versus negative groups.

### 3.2. Incidence of DMRGM

Overall, we found DMRGM in 30.7% patients. Twenty-six patients had one mutation, eight patients had two, and one patient had three mutations, with no cases of four or more mutations. Among ELN favorable risk patients (n = 37), a total of 21 DMRGM occurred in 16 (43.2%) patients: 21.6% with *DNMT3A*; 13.5% with *IDH2*; 13.5% with *TET2* and 8.1% with *IDH1* mutation. Among the ELN intermediate risk group (n = 38), a total of 16 DMRGM occurred in 12 (31.6%) patients: 13.2% with *DNMT3A*; 7.9% with *IDH2*; 13.2% with *TET2* and 7.9% with *IDH1* mutation. Within the ELN adverse risk (n = 39) group, a total of eight DMRGM occurred in seven (17.9%) patients: 7.7% with *DNMT3A*; 5.1% with *IDH2*; 2.6% with *TET2* and no patients with IDH mutation, and *SETBP1* 5.1% (2/39). Table 2 summarizes the incidence of DMRGM in our cohort. These findings led to 16, 21, 12, 26, 7 and 32 patients classified as groups 1, 2, 3, 4, 5 and 6, respectively. DMRGM occurred more frequently in the ELN favorable group (16/37; 43.2%) than the adverse group (7/39, 17.9%) (*p* = 0.0164). However, the rates of DMRGM were not statistically significantly different between the favorable and intermediate groups or between the intermediate and adverse groups.

### 3.3. Survival Analysis

**Figure 1:**
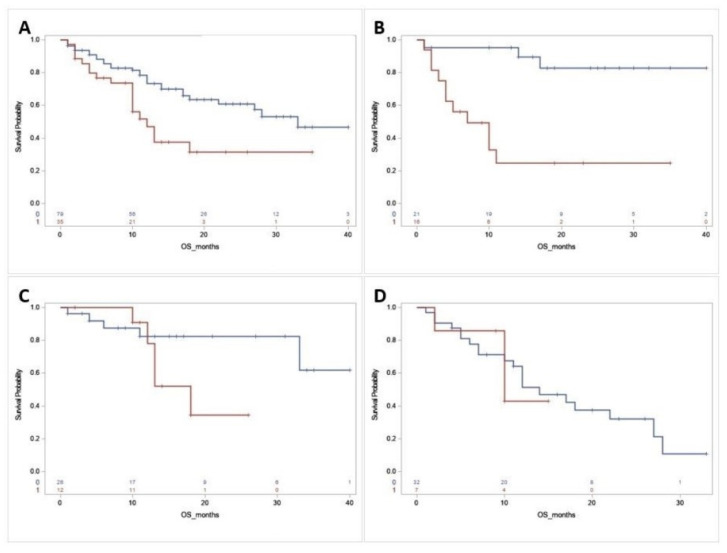
Overall survival (OS) rates by DNA methylation mutation. Red Line: DNA methylation regulatory gene (DMRGM) mutation positive. Blue Line: DMRGM negative. (**A**) OS rate for all AML cases by DMRGM positive versus negative (medial OS: 12 months vs. 33 months, *p =* 0.0053). (**B**) OS rate for 2017 ELN favorable group by DMRGM positive versus negative (median OS: 7 months vs. not reached, *p* = 0.0001). (**C**) OS rate for 2017 ELN intermediate group by DMRGM positive versus negative (*p =* 0.1172). (**D**) OS rate for 2017 ELN adverse group by DMRGM positive versus negative (*p =* 0.7773).

Among the entire cohort of patients, the OS of patients with DMRGM was significantly worse than that of patients without DMRGM (medial OS: 12 months vs. 33 months, *p =* 0.0053, Figure 1A). Multivariate analysis showed that DMRGM status was an independent unfavorable prognostic factor for OS (hazard ratio (HR): 2.704, 95% confidence interval (CI): 1.451–5.041, *p =* 0.0017, Table 3a).

**Figure 2:**
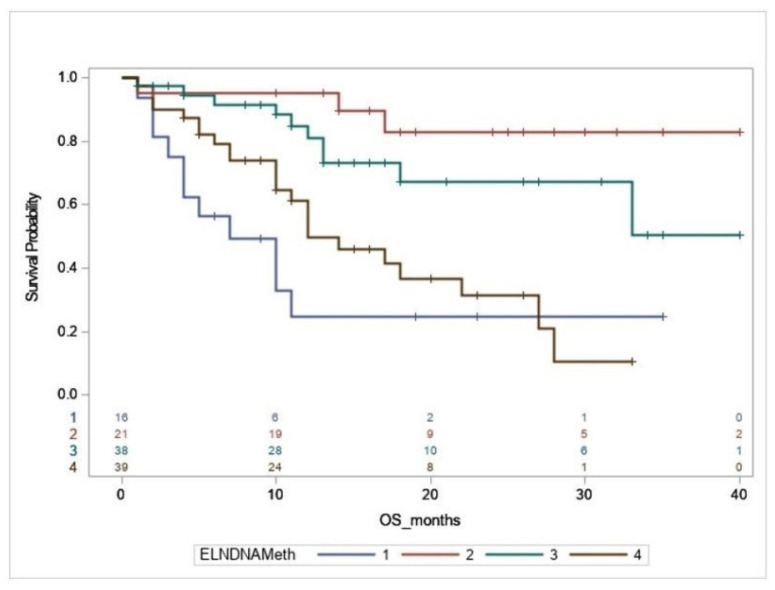
Overall survival (OS) rates in ELN favorable group with and without DNA-MR, ELN intermediate, and ELN adverse group. Kaplan–Meier curves for OS in ELN favorable with DMRGM (**Blue** line: 1, median OS: 7 months), ELN favorable without DMRGM (**Red** line: 2, median OS: Not reached), ELN intermediate (**Green** line: 3, median OS: Not reached) and ELN adverse group (**Brown** line: 4, median OS: 12 months). The OS of the ELN favorable with DMRGM was significantly shorter than that of the favorable group without DMRGM (*p =* 0.0001) or the ELN intermediate group (*p =* 0.0078). The median OS of ELN favorable group with DMRGM was not significantly different from that of ELN adverse group (*p =* 0.9937).

We subsequently examined the impact of DMRGM on survival within each 2017 ELN risk group. For the ELN favorable group (n = 37), the median OS of patients with DMRGM was significantly shorter than those without DMRGM (7 months vs. not reached, *p =* 0.0001, Figure 1B). Of note, among the ELN favorable group, patients with mutated *NPM1 I* (n = 21) DMRGM showed similar impact on median OS (DMRGM vs. no DMRGM: 7 months vs. not reached, *p* = 0.016). In multivariate analysis, the DMRGM status remained an independent unfavorable prognostic factor for OS (HR: 6.882, 95% CI: 1.24–38.184, *p =* 0.0274, Table 3b). In contrast, DMRGM showed no significant impact on OS in either the ELN intermediate group or ELN adverse group (Figure 1C,D). Of importance, the median OS of the ELN favorable group with DMRGM was significantly shorter than that of the ELN intermediate group (7 months vs. not reached, *p =* 0.0078, Figure 2) and also appeared shorter than that of the ELN adverse group, although not statistically significant (7 vs. 12 months, *p =* 0.9937, Figure 2).

### 3.4. Genetic Association of DMRGM with Other Mutations in the 2017 ELN Favorable Risk Group

**Figure 3:**
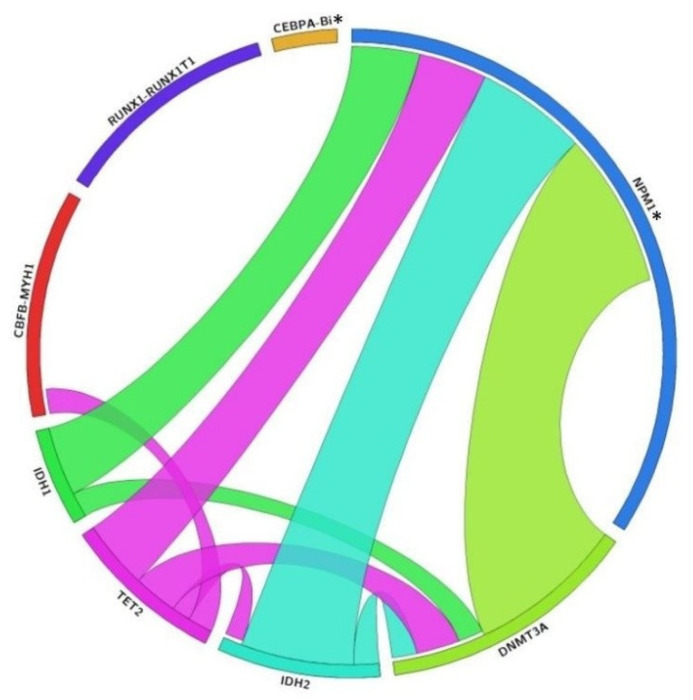
Mutational profile of 2017 favorable AML group in our cohort by using Circos plot model. The thickness of connection for each mutation is proportional to the number of co-occurring cases. Among 2017 favorable mutations, only *NPM1* mutation significantly co-occurred with DNA methylation-related gene mutations (including *DNMT3A*, *TET2*, *IDH1*, and *IDH2*). * Abbreviation: CEBPA-Bi; Biallelic mutated *CEBPA*, NPM1; Mutated *NPM1* without *FLT3*-ITD or with *FLT3*-ITD^low^.

Among the ELN favorable group with DMRGM (n = 16), DMRGM occurred predominantly in cases with mutated *NPM1* (15/16, or 93.8%, Figure 3). The remaining one case concurred with CBFB-MYH1 fusion (Figure 3). The DMRGM in mutated *NPM1* cases appeared independent of *FLT3*-ITD mutations (eight patients had FLT3-ITD^low^, and seven patients were without *FLT3*-ITD).

## 4. Discussion

The results of this study indicate that DMRGM commonly occur in AML and they adversely impact the prognosis of AML patients based on the 2017 ELN risk classification. The impact of these mutations was mainly seen in the favorable risk group, particularly those with *NPM1* mutation, while they had no impact on the ELN intermediate or adverse risk category.

Although the prognostic impact of DMRGM as a group of genes on AML patients has not been extensively studied, multiple studies on individual genes involving DNA methylation regulations correlate with our findings. Several studies suggest that *DNMT3A* adversely impacts OS effect on AML patients [6,14,15]. In a meta-analysis of 4500 AML patients by Shivarov et al., *DNMT3A* mutations showed a significantly worse prognosis, particularly in patients with cytogenetically normal AML (CN-AML) [29]. *IDH1/2* mutations have also been shown to adversely impact OS in AML patients [16,17]. In a recent meta-analysis, *IDH1/2* mutations showed no OS affect in the whole AML population; however, *IDH1* mutation conferred a worse OS in patients with CN-AML (OS: HR, 1.21; 95% CI, 1.01–1.46) [30]. Additionally, similar to our findings, others have shown that IDH mutations tended to occur with *NPM1* but not *FLT3*-ITD mutations [5,17]. Furthermore, some studies reported that the negative impact of *IDH 1* and *2* mutations was only seen in mutated *NPM1* with wild-type *FLT3*, but not in other AML patients [17,31]. With respect to *TET2* mutations, a meta-analysis by Liu et al. in 2552 AML patients showed that it was associated as an adverse prognostic indicator only in CN-AML (OS: HR, 1.43, 95% CI: 1.16–1.75, *p =* 0.001) [32]. Tian et al. evaluated the impact of *TET2* in 373 adults with CN-AML patients. They reported that *TET2* mutation was an unfavorable prognostic factor leading to shorter median OS as compared to wild-type *TET2*, particularly in *FLT3*-ITD negative, *NPM1* positive patients (9.5 vs. 32.2 months, *p =* 0.013) [33]. Though it is well established that mutated *NPM1* has favorable outcomes in AML, our results and the above studies suggest that DMRGM may adversely impact the OS of patients with mutated *NPM1*. Although many of the above studies considered the effects of cytogenetic findings and *NPM1* mutation, none of these studies specifically looked into the impact of these gene mutations among different ELN risk groups.

Our approach of considering DMRGM as a group is similar to the study by Ryotokuji et al., in which the investigators integrated four genes (*IDH1/2*, *TET2* and *DNMT3A*) involving DNA methylation regulation as a group and analyzed its prognostic impact on AML. In their study, cases with DMRGM had a significantly poorer OS than those without DMRGM in all cases of AML, in agreement with our findings [24]. However, this study did not evaluate the prognostic effect of DMRGM by ELN risk stratification. Our study further found that the adverse impact of DMRGM on AML patients was only in patients in the ELN favorable risk group. However, the mechanisms leading to the adverse impact remain unclear and require future studies. Of note, the adverse impact appears not associated with the complete response (CR) rate. In the entire cohort, the CR rate of patients with DMRGM was not different from the patients without DMRGM. Similarly, DMRGM status did not impact the CR rate within each ELN risk group.

In contrast to our observation, some studies have shown that mutations of DNA methylation regulatory genes may not impact the prognosis of AML patients. For example, Gaidzik et al. reported no significant survival impact of *DNMT3A* mutation in a cohort of 1770 adult AML patients [19]. Shen et al. stated that *DNMT3A*, but not *IDH1/2*, mutations adversely impacted OS in a cohort of 605 AML patients [20]. Moreover, Mason et al. reported that *NPM1*-mutated AML patients with *TET2*, *IDH1/2* mutations had better OS than *NPM1*-mutated patients without such mutations [34]. The mechanisms leading to these discrepancies remain uncertain. Further studies with larger sample sizes are needed to fully illustrate the prognostic impact of DMRGM in AML.

Grouping DMRGM as a functional group of genes to study the impact on the survival of AML patients can be controversial. For example, *SETBP1* mutations in AML are relatively uncommon compared to *TET2*, *IDH1/2* and *DNMT3A*, and the prognostic impact of *SETBP1* mutation has not been well documented. However, it is well established that *IDH1*, *IDH2* and *TET2* are usually mutually exclusive in AML [5,18,22,35]. In addition, mutations of these three genes have similar epigenetic impacts leading to global DNA hypermethylation [22,36,37,38]. *DNMT3A* has also been reported to play an important role in DNA methylation, and several studies have demonstrated a correlation between *DNMT3A* mutation and DNA methylation, although the function and biological consequences of *DNMT3A* mutations have yet to be fully elucidated [38,39,40]. Additionally, particular mutations of *DNMT3A* may have different effects on methylation regulation. Some studies have suggested that *SETBP1* is involved in methylation [41,42]. In the current study, only two patients had *SETBP1* mutations, both in the ELN adverse risk group, and DMRGM did not impact survival outcome in this group. Future studies, particularly for *SETBP1*-mutated patients, are needed to validate the approach of considering DMRGM as a functional group.

Among the ELN favorable risk group, the cases with mutated *NPM1* show strong concurrence with DMRGM in our study. This finding agrees with previous studies which have reported that *DNMA3A*, *IDH1/2* and/or *TET2* frequently co-occurred with *NPM1* mutation in AML patients [4,5,43]. The study by Papaemmanuil et al. revealed that 73% (319 of 436) of *NPM1*-mutated AML patients are positive for mutations in at least one of the following genes: *DNMT3A*, *IDH1*, *IDH2*, and *TET2* [5]. Our findings confirm that DMRGM frequently concur with *NPM1* mutation and suggest these mutations may adversely impact OS.

Although our study suffers from limitations of small sample size, particularly only 37 patients in the ELN favorable group with 16 of them carrying DMRGM, and retrospective analysis, it can have important clinical implications if confirmed by others. The current recommendation is to advise against allogeneic stem cell transplantation in patients with favorable risk disease in CR1. Thus, ELN favorable risk patients, particularly those defined by mutated *NPM1*, with DMRGM may not receive adequate treatment. Our findings suggest the importance of including DNA methylation regulatory genes in the targeted NGS panel along with the genes used to define current ELN risk groups to further evaluate the clinical significance of DMRGM.

## 5. Conclusions

In conclusion, our results indicate that DMRGM are common in AML patients and adversely impact the prognosis of AML patients, particularly those in the 2017 ELN favorable risk group defined by mutated *NPM1*. The latter patients had OS similar to that of patients in the ELN adverse risk group. Further studies with a large sample size are warranted to confirm our observations.

## Figures and Tables

**Table 1:** Clinical/laboratory characteristics and AML classification of patients according to DNA methylation regulatory gene mutation status.

Characteristic	All Patients (n = 114)	DMRGM * Positive (n = 35)	DMRGM Negative (n = 79)	*p*-Value
**Age, years**				0.0748
Median	61.5	63.16	58.92	
IQR *	(48.44, 67.11)	(56.29, 69.31)	(46.52, 66.85)	
**Sex, No. (%)**				0.1859
Male	69 (60.53)	18 (51.43)	51 (64.56)	
Female	45 (39.47)	17 (48.57)	28 (35.44)	
**WBC, 10^3^/uL**				0.3363
Median	7.5	11.57	7.05	
IQR	(2, 37.57)	(2.1, 55.8)	(1.93, 30)	
Missing Values	0	0	0	
**BM blasts (%)**				0.8825
Median	51	58	47.6	
IQR	(25, 72)	(25, 73.2)	(25, 69.2)	
Missing values	0	0	0	
**PB blasts (%)**				0.2977
Median	18	18	18	
IQR	(1, 60)	(0, 60)	(2, 60)	
Missing values	8	2	6	
**Platelet counts, 10^3^/uL**				0.303
Median	52	53.5	52	
IQR	(24, 89)	(29, 100)	(23, 89)	
Missing values	1	1	0	
**Hemoglobin, g/dL**				0.3195
Median	8	8.2	7.9	
QR	(7, 9.5)	(7.2, 9.5)	(6.5, 9.5)	
Missing values	0	0	0	
**2017 ELN risk,** **No. (%)**				0.057
Favorable	37 (32.46)	16 (45.71)	21 (26.58)	
Intermediate	38 (33.33)	12 (34.29)	26 (32.92)	
Adverse	39 (34.21)	7 (20.00)	32 (40.50)	
HSCT *, No. (%)				0.4019
Not Received	65 (57.02)	22 (62.86)	43 (54.43)	
Received	49 (42.98)	13 (37.14)	36 (45.57)	
**Classification of AML, No. (%)**				
AML with t(8;21)(q22;q22.1);RUNX1-RUNX1T1	7 (6.14%)	0 (0.00%)	7 (8.86%)	
AML with inv(16)(p13.1q22) or t(16;16)(p13.1;q22);CBFB-MYH11	7 (6.14%)	1 (2.86%)	6 (7.59%)	
AML with t(9;11)(p21.3;q23.3);MLLT3-KMT2A	2 (1.75%)	2 (5.71%)	0 (0.00%)	
AML with mutated *NPM1*	25 (21.93%)	16 (45.71%)	9 (11.39%)	
AML with biallelic mutations of *CEBPA*	2 (1.75%)	0 (0.00%)	2 (2.53%)	
AML with myelodysplasia-related changes	31 (27.19%)	9 (25.71%)	22 (27.85%)	
AML, NOS	40 (35.09%)	7 (20.00%)	33 (41.77%)	

* Abbreviation: DMRGM; DNA methylation regulatory gene mutations, HSCT; Hematopoietic stem cell transplantation, IQR; Interquartile range, NO.; Number.

**Table 2:** Incidence of DNA methylation regulatory gene mutations in the whole cohort and among ELN risk groups.

	*DNMT3A*	*IDH1*	*IDH2*	*TET2*	*SETBP1*
Whole AML cohort (n = 114)	14.0% (16/114)	5.3% (6/114)	8.8% (10/114)	9.6% (11/114)	1.8% (2/114)
Favorable risk (n = 37)	21.6% (8/37)	8.1% (3/37)	13.5% (5/37)	13.5% (5/37)	0.0% (0/37)
Intermediate risk (n = 38)	13.2% (5/38)	7.9% (3/38)	7.9% (3/38)	13.2% (5/38)	0.0% (0/38)
Adverse risk (n = 39)	7.7% (3/39)	0.0% (0/39)	5.1% (2/39)	2.6% (1/39)	5.1% (2/39)

**Table 3:** Multivariate analysis of prognostic factors for overall survival.

**(a) Cox hazard proportional models in all AML patients (n = 114)**				
**Variables**		HR *	95% CI *	***p*-Value**
DMRGM *	Yes	2.704	(1.451, 5.041)	0.0017
	(Reference, No)			
2017 ELN risk	Intermediate	0.93	(0.392, 2.208)	0.8693
	High	2.911	(1.379, 6.186)	0.0051
	(Reference, Favor)			
Age	65 and over	1.124	(0.592, 2.135)	0.7205
	(Reference, under 65)			
HSCT *	Yes	0.37	(0.19, 0.719)	0.0034
	(Reference, No)			
**(b) Cox hazard proportional models for 2017 ELN favor risk group (n = 37)**				
**Variables**		**HR**	**95% CI**	***p*-Value**
DMRGM	Yes	6.882	(1.24, 38.184)	0.0274
	(Reference, No)			
Age	65 and over	1.235	(0.314, 4.857)	0.763
	(Reference, under 65)			
HSCT	Yes	0.534	(0.161, 1.77)	0.3049
	(Reference, No)			
*NPM1* Mutation	Yes	1.629	(0.251, 10.589)	0.6092
	(Reference No)			
*FLT3*-ITD^Low^	Yes	1.188	(0.363, 3.891)	0.7761
Mutation	(Reference No)			

* Abbreviation: CI; Confidence interval, DMRGM; DNA methylation regulatory gene mutations, HR; Hazard ratio, HSCT; Hematopoietic stem cell transplantation.

## Data Availability

Not applicable.
